# Crimean-Congo Hemorrhagic Fever Virus among Goats, Southern Bhutan

**DOI:** 10.3201/eid3108.241989

**Published:** 2025-08

**Authors:** Sangay Rinchen, Yoshihiro Kaku, Eunsil Park, Puspa Maya Sharma, Dawa Tshering, Tenzinla Tenzin, Aya Matsuu, Akiko Okutani, Ken Maeda, Shigeru Morikawa

**Affiliations:** National Centre for Animal Health, Serbithang, Bhutan (S. Rinchen, P.M. Sharma, D. Tschering, T. Tenzin); National Institute of Infectious Diseases, Shinjuku, Tokyo, Japan (Y. Kaku, E. Park, A. Matsuu, A. Okutani, K. Maeda, S. Morikawa)

**Keywords:** Crimean-Congo hemorrhagic fever virus, viruses, CCHFV, antibodies, goats, Bhutan

## Abstract

We conducted serosurveillance for Crimean-Congo hemorrhagic fever virus (CCHFV) among goats in southern Bhutan. Testing serum samples from 472 goats for CCHFV-specific IgG using an indirect fluorescent antibody test and ELISA, we found CCHFV antibody–positive goats along the analyzed border region with India, indicating widespread distribution of CCHFV in this area.

Crimean-Congo hemorrhagic fever (CCHF) is an acute febrile zoonosis caused by CCHF virus (CCHFV). CCHFV belongs to the genus *Orthonairovirus* (family Nairoviridae, order Bunyavirales) (*1*). It is transmitted by ticks, particularly of the genus *Hyalomma*, and is widespread across Africa, Asia, and Europe. Symptoms associated with CCHFV include fever, headache, myalgia, back pain, and arthralgia, and infected patients demonstrate varying degrees of hemorrhage (petechiae to maculopapular) in severe cases. Ticks play a crucial role in the CCHFV infection cycle, maintaining the virus through transstadial and transovarial transmission, and the virus persists in a tick–vertebrate–tick enzootic cycle. Human infection can result from tick bites or direct contact with asymptomatic animals, and CCHF occurs most frequently among livestock workers, slaughterhouse workers, and veterinarians. To mitigate the infection risk in humans, a One Health approach, including proactive surveillance of animals and ticks, is crucial.

In India, researchers described the first reported human case of CCHF in Gujarat in 2011; subsequent reports documented sporadic outbreaks (*2*–*4*). Since those initial reports, investigators have conducted CCHFV infection surveys in livestock and ticks in various locations in India, noting the virus’ widespread distribution (*5*–*7*). 

Bhutan shares a long and porous border with India, providing many opportunities for animal and human movement between countries. Continuous surveillance of various zoonotic agents in this area is therefore critical in assessing the risk for infection to animals and humans. To investigate CCHFV, a research team conducted a pilot serosurvey in 2015 using livestock sera collected in the southern region of Bhutan (*8*). They collected 81 goat samples from Sarpang district and 92 bovine samples from Trashigang and Samtse districts and tested them for CCHFV-specific IgG using an in-house ELISA kit (National Institute of Virology, Pune, India). Unfortunately, the results of this pilot survey did not fully elucidate the seroprevalence of CCHFV in southern Bhutan because of the limited sample size and study area. 

To obtain more detailed information on the geographic range of CCHFV antibody-positive animals in southern Bhutan, we focused our study on goats in the southern border region, particularly the western and central areas along the border, where multiple cross-border animal trade hubs exist. We analyzed a total of 472 goat serum samples, collected in 2015 and 2022 from those border areas, using a combination of 2 testing methods: a different in-house ELISA kit (National Institute of Infectious Diseases, Tokyo, Japan) and an indirect fluorescent antibody test (Appendix). We employed this dual analytical approach to improve specificity against CCHFV antibodies, considering the possibility that multiple *Orthonairovirus* species co-circulate.

We charted seroprevalence of CCHFV in each district (Table), noting the presence of CCHFV antibody–positive goats in all surveyed districts from the central to western parts of Bhutan’s southern border region. Our results confirmed the widespread seropositivity of CCHFV in this region, also revealing substantial regional variation in antibody positivity, ranging from high-positive (Sarpang, Samtse, and Chukha) to low-positive (Dagana and Tsirang). The 3 districts with high seropositivity rates are among the key formal entry points from India to Bhutan, characterized by numerous cross-border settlements, robust trade activities, and fluid cross-border movement of humans and animals. Considering both sides of the border as the same epidemiologic unit, the detection of seropositive animals in multiple districts suggested that CCHFV circulates in this region. 

**Table:** Comparison of viral seroprevalence among goats in southern border region in study of Crimean-Congo hemorrhagic fever virus among goats, southern Bhutan

District	No. tested samples	No. positive samples	Seroprevalence, %	Year of sample collection
Samtse	153	67	43.8	2015
Sarpang	81	49	65.1	2015
Chukha	123	39	31.7	2022
Dagana	74	6	8.1	2022
Tsirang	41	2	4.9	2022

Our initial plan for this study entailed collecting samples in a much shorter timeframe; however, budget constraints and the COVID-19 outbreak resulted in a longer time lag between collection years. Nonetheless, considering the frequent cross-border movement of animals and humans and the lack of comprehensive tick control measures in southern Bhutan, we postulated that the seroprevalence in goats did not change considerably during or after the sampling period. A better understanding of the spatial and temporal patterns of viral distribution of CCHFV in this region of Bhutan will require a longitudinal study targeting a larger sample size of animals and ticks.

Our findings document widespread seropositivity to CCHFV in goats in the western and central regions along Bhutan’s southern border. In a previous serologic study (*8*), CCHFV-specific IgG was not detected in bovine samples from Samtse and Trashigang districts, which might have been a result of the limited sample size and study area. Cattle are known to be susceptible to CCHFV infection, so further testing of additional bovine samples is necessary to investigate CCHFV infection among cattle in Bhutan. More research is also needed to collect and analyze ticks to investigate their viral infection status. By obtaining viral genetic information from ticks in this region of Bhutan, researchers can confirm the genotypes of CCHFV prevalent in this area, providing potential insight into virus circulation. Because human CCHF cases might be underreported in Bhutan, conducting antibody testing of livestock workers in the country’s southern region might inform both prevalence data and public health initiatives to educate workers on preventive measures to protect against CCHFV infection.

Appendix Additional information for Crimean-Congo hemorrhagic fever virus among goats, southern Bhutan.
