# Supplementary material for: Crimean-Congo Hemorrhagic Fever Virus among Goats, Southern Bhutan
Source: Emerg Infect Dis. 2025 Aug;31(8):1657–8. doi: 10.3201/eid3108.241989 (PMC12309775; doi:10.3201/eid3108.241989)
Supplement: Appendix — Additional information for Crimean-Congo hemorrhagic fever virus among goats, southern Bhutan. [file 24-1989-Techapp-s1.pdf]

Article DOI: http://doi.org/10.3201/eid3108.241989

EID cannot ensure accessibility for supplementary materials supplied by authors. Readers who have difficulty accessing supplementary content should contact the authors for assistance.

Crimean-Congo Hemorrhagic Fever Virus among Goats, Southern Bhutan

Appendix

Sample collection

The collection year of the goat serum samples and the five districts ("Dsonkhag" in Bhutanese) of Bhutan included in this study are outlined in the Table and in the Appendix Figure. The goat serum samples obtained in 2015 (in Samtse and Sarpang) were collected initially as part of a survey on peste des petits ruminants and were used in this study with permission from the Department of Livestock (DoL), Ministry of Agriculture and Livestock (MoAL), Bhutan. The collection of goat serum samples in 2022 (in Chukha, Dagana, and Tsirang) and the implementation of this study were approved by the Livestock Technical Advisory Committee of the DoL, MoAL, Bhutan, under Approval number DoL/GEN-03/2020–2021/108, dated 24/05/2021. In both sample collections, the target areas were mainly set as sub-districts ("Gewog") near the national borders of each district, and as many villages as possible were selected from within each sub-district. A total of 472 goat serum samples were collected from 96 farms that cooperated in this study, located in 54 villages, 22 sub-districts, and five districts. The number of samples collected per farm ranged from 1 to 44, with a median

of 2.5 samples. Limitations, such as an insufficient number of farms and total livestock by region for statistical analysis and difficulty obtaining consent from all farms, hindered the implementation of a comprehensive study design and uniform criteria that reflected the animal population.

Goats below 6 months of age were excluded from the study because of concerns regarding bleeding young animals. Approximately 8 mL of blood was collected from the jugular vein of each of the selected animals. Serum was separated from the whole blood samples and was transported at -20°C to the National Veterinary Laboratory at the National Centre for Animal Health, Thimphu, where it was stored at -20°C before testing.

Testing methods

An ELISA targeting the nucleoprotein (NP) of CCHFV expressed in a baculovirus system was performed as previously described (*1*). First, the sensitivity and specificity of the ELISA were calculated using half of the samples by creating receiver operating characteristic (ROC) and 2-graph ROC curves using StatFlex version 5 software (*2*). To confirm the positive ELISA results, an indirect fluorescent antibody test (IFAT) with HeLa cells expressing recombinant CCHFV NP antigens was performed using 1:50-diluted serum samples as previously described (*1*). IFAT positivity was examined using fluorescence microscopy. The distribution of OD values for the ELISA was compared for IFAT-positive and IFAT-negative samples. The area under the ROC was calculated in the respective groups and indicated that the ELISA was able to discriminate IFAT-positive from IFAT-negative samples with high probability. The cutoff value was set to provide optimal sensitivity and specificity. The

remaining serum samples were also subjected to ELISA and analyzed using the cutoff value.

Reporting guidelines for research

This study was reported in accordance with the STROBE Guidelines (STrengthening the Reporting of OBservational studies in Epidemiology) for observational studies, and statistical methods were described in line with the SAMPL Guidelines (Statistical Analyses and Methods in the Published Literature).

References

1. Oluwayelu D, Afrough B, Adebiyi A, Varghese A, Eun-Sil P, Fukushi S, et al. Prevalence of Antibodies to Crimean-Congo Hemorrhagic Fever Virus in Ruminants, Nigeria, 2015. Emerg Infect Dis. 2020;26:744–7. PubMed https://doi.org/10.3201/eid2604.190354

2. Greiner M, Sohr D, Göbel P. A modified ROC analysis for the selection of cut-off values and the definition of intermediate results of serodiagnostic tests. J Immunol Methods. 1995;185:123–32. PubMed https://doi.org/10.1016/0022-1759(95)00121-P

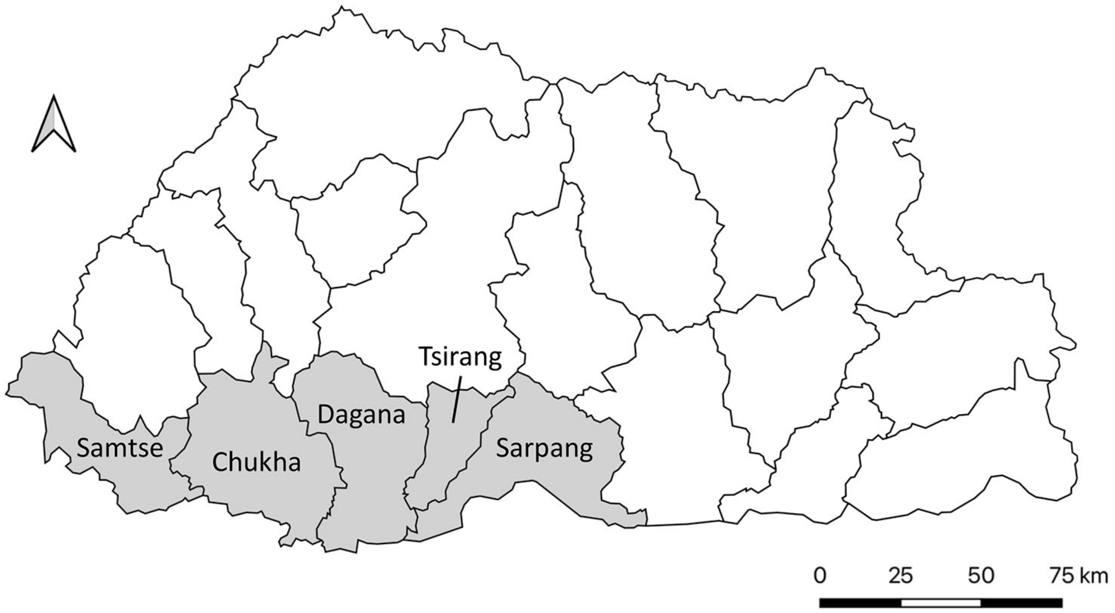

Appendix Figure. Districts of Bhutan included in a study of Crimean-Congo hemorrhagic fever virus among goats, southern Bhutan. Shaded areas indicate districts ("Dsonkhag") of Bhutan in which serum samples were collected from goats and tested for antibodies against Crimean-Congo hemorrhagic fever virus (CCHFV, *Orthonairovirus hemorrhagiae*).
